# Management of High-Grade Penile Curvature Associated With Hypospadias in Children

**DOI:** 10.3389/fped.2017.00189

**Published:** 2017-09-04

**Authors:** Paulo R. M. Moscardi, Rafael Gosalbez, Miguel Alfedo Castellan

**Affiliations:** ^1^Pediatric Urology, Children’s Urology Associates, Miami, FL, United States

**Keywords:** penile curvature, hypospadias, review of literature, children, high-grade curvature

## Abstract

Penile curvature is a frequent feature associated with hypospadias with also a great variability of severity among each patient. While the low-grade curvature (<30°) can be relatively easily corrected by simple techniques like penile degloving and dorsal plication, severe cases often demand more complex maneuvers to manage it. A great number of surgical techniques have been developed to adequately correct curvatures greater than 30°; however, each one of them should be individualized to different patients and local conditions encountered. In this article, we will review the evaluation of the pediatric patient with penile curvature associated with hypospadias with a special attention to high-grade cases, their management, indications for surgical treatment, and several surgical options for their definitive treatment.

## Introduction

Penile curvature is a broad-spectrum disorder usually not only associated with hypospadias but also encountered in children with normal meatus. The prevalence of hypospadias is roughly 1 in 300 male births; of these, 1 in 4 will have some degree of curvature (1). Penile curvature can also be seen in children without hypospadias with the diagnosis being delayed most of the time in this subset of patients. It is believed that the real incidence of penile curvature is around 4–10% of male births (2).

Donnahoo et al. reported a large series of patients with congenital penile curvature without hypospadias and grouped these cases according to the etiology of the curvature, which comprised skin tethering, fibrotic dartos and Buck’s fasciae, corporeal disproportion, and urethral tethering. Among the 87 patients included 84, 11, and 5% had ventral, dorsal, and lateral curvatures, respectively (3).

A number of theories have been proposed to explain the origin of penile curvature such as abnormal development of the urethral plate (UP), fibrotic mesenchymal tissue at the urethral meatus, and ventral–dorsal corporal disproportion (1, 4). During 16th week of the fetal period, the penis has a physiologic ventral curvature (VC), with this resolving between the 20th and 25th week (4). An arrest of the embryonic development at some point during this process maintains this curvature. Moreover, the occurrence of the urethral defect is associated with thinning and hypodevelopment of the corpus spongiosum and other ventral structures that ultimately leads to penile disproportion and curvature. All of these factors may be interconnected and have different roles and degrees of influence on the final severity of curvature in each patient. This objectively affects surgical management requiring individualized treatment for each patient. In addition, the neurovascular anatomy is also altered with the neural supply originating under the pubic rami running superior and lateral to the urethra, leaving the dorsal aspect of the penis bereft of neural tissue (5).

In this article, we will review the evaluation of the pediatric patient with penile curvature associated with hypospadias with a special attention to high-grade cases, their management, indications for surgical treatment, and several surgical options for their definitive treatment.

## Evaluation of Abnormal Penile Curvature in Children

The association of hypospadias and penile curvature in children requires a comprehensive evaluation including measuring the degree of curvature as well as its direction and assessment of other genital abnormalities like urethral hypoplasia, cryptorchidism, and penoscrotal transposition.

The feasibility of penile curvature evaluation during an outpatient consult is limited. The degree of curvature is properly evaluated while the penis is full in erection at the time of physical examination. Unfortunately, this is not a common event during the evaluation of these patients. Consequently, the reliability of curvature assessment should be considered only at the time of the surgery.

A high percentage of penile curvatures are resolved just by degloving the penis, the shortened ventral skin, and fibrotic ventral dartos at the beginning of the hypospadias surgery. These surgical steps can resolve the curvature with no need of a further treatment.

Braga et al. reviewed the resolution rate of the penile curvature after the dissection of the skin and the dartos fascia in patients with hypospadias (6). In this study, 137 patients had their curvature evaluated at the start of the procedure, and 9 had mild (<30°), 44 moderate (30–45°), and 85 severe curvature (>45°). The complete resolution rate of the curvature, just after degloving the penis per group, was 70% in the mild, 30% in the moderate, and 2.4% in the severe curvature.

The method most frequently used to measure the degree of curvature is the Gittes and McLaughlin technique (7). An artificial erection is fashioned by injecting saline through a fine needle (25–27 gauge) into the lateral aspect of the corpora cavernosa or into the glans with a tourniquet at the base of the penis. Consequently, the degree of curvature must be evaluated through an artificial erection after degloving the penis and dissection of the skin and the fibrotic dartos fascia.

## What Degree of Penile Curvature is Significant?

Measuring the level of penile curvature is crucial before its surgical correction, since a low degree of physiological curvature usually do not imply a necessity for correction. It is believed that a curvature greater than 20–25° found in children is significant and should be corrected.

Bologna et al. in 1999 reported a survey among pediatric urologists members of the American Academy of Pediatrics regarding the treatment of penile curvature in patients with hypospadias (8). No intervention on the curvature was preferred in 92, 25, and <1% when the curvature was 10°, 20°, and 30°, respectively. More recently, a similar survey has shown an even more conservative trend. No intervention was chosen among the respondents in 69, 64, and 16% when the curvature was 10°, 20°, and 30°, respectively. Furthermore, in this later survey, the dorsal approach (simple plication or Nesbit procedure) was the procedure of choice even when for a high-grade curvature, with these techniques being the procedure of choice in 66 and 47% when degree of curvature was 40° and 50°, respectively (9).

However, the significance of the degree of curvature may be perceived differently by patients and parents. Currently, there are no studies that address specifically in terms of curvature what is significant or not for patients/parents. Usually this assessment is done in the postoperative period. Fraumann et al. (10) evaluated the long-term outcomes on adults who underwent severe hypospadias repair during childhood. Of the 13 patients, 4 (31.0%) reported curvature <30° and 1 reported curvature >30° Overall, 11 men (85.0%) were satisfied with their penile appearance. Despite persistent curvature, only two patients described the overall penile appearance as “somewhat unsatisfactory” or “very unsatisfactory.” Moreover, the significance of the clinical degree of curvature later in life should be carefully analyzed since the relevance of the curvature is subjective from patient to patient.

## Low-Grade Penile Curvature

A broad spectrum of severity and prevalence of the VC is seen in patients with hypospadias. The frequency of penile curvature in distal hypospadias patients have been reported among 3–33% of patients after dissection of penile skin and the dartos fascia (11) with the majority being of low grade (less than 30°).

Even in patients with proximal hypospadias, a high prevalence of low-grade curvature is seen. Snodgrass and Prieto reported an absence or mild curvature (<30°) in 50% of patients with proximal shaft to perineal hypospadias (12). Among these, 31% needed a simple dorsal plication to correct the curvature.

## High-Grade Penile Curvature

Proximal hypospadias associated with high-grade curvature (>30°) occurs in approximately 5–10% of the cases; the management of these complex cases represents a challenge to the pediatric urologist, being this the group with the highest number of reoperations and severe complications. A summary of the main studies reported on the literature are shown in Table 1.

**Table 1:** Proximal hypospadias/VC repair.

Study	No of patients	Mean follow-up (months)	Meatal location	Repair type	VC correction techniques used	Total complications	Residual curvature
Ghali et al. (13)	148	23	Midshaft/proximal	Tubularized preputial flap	Degloving/UP mobilization/DA^a^	32%	2%
Ferro et al. (14)	34	1–48 (range)	Proximal	2-stage preputial graft	Degloving/DA	24%	0
Johal et al. (15)	62	26 (median)	Midshaft/proximal	2-stage grafts	Degloving/UP transection/DA	18%	5%
Braga et al. (16)	100	65	Proximal	Transverse preputial flap	DP 68	68%	27.9%
					VLG 32	43.7%	9.4%
Ghanem and Nijman (17)	49	36	Proximal	TIP	Degloving/DA	12%	0
Snodgrass and Bush (18)	36	12	Proximal	TIP	UP mobilization/DA/ventral corporotomies w/o corporal grafts^b^	13%	0
McNamara et al. (19)	134	45.6 (median)	Proximal	2-stage flap	Degloving	53%	2%
					DP		
					Extensive ventral dissection		
					VL w/grafts		
Pippi Salle et al. (20)	140	TIP (48.3); DIG (35.7); 2-stage (29.6)	Proximal	TIP/DIG/2-stage	TIP/DIG: UP mobilization/DA + UP section in DIG cases	TIP: 61.4%	TIP: 14%
					2-stage: DTITA ± DA in the second stage	DIG: 52.1%	DIG: 17%
						2-stage: 38.3%	2-stage: 5%
Chen et al. (21)	87	TPFI: 38 (median)	Proximal	Staged TPIF/2-stage Byars urethroplasty	Degloving; DA; UP transection	TPFI: 9.5%	0
		Byars: 36 (median)				Byars: 33%	
Long et al. (22)	167	31.7 (median)	Proximal	1-stage	DP/ventral “fairy” cuts/VLG	1-stage: 62%	1-stage: 7%
				2-stage		2-stage: 49%	2-stage: 3.7%
Snodgrass and Bush (23)	43	22	Proximal	2-stage	Corporotomies w/o corporal grafts at the 1-stage	23%	0
Lanciotti et al. (24)	50	63.6	Proximal	2-stage (bladder graft)	Degloving/DA	46%	10%

*UP, urethral plate; DIG, dorsal inlay graft; DTITA, deep transverse incisions of tunica albuginea; DP, dorsal approach (plication/Nesbit); TPIF, transverse preputial island flap; VLG, ventral lengthening w/grafts; VC, ventral curvature*.

*^a^UP was divided in severe VC cases in the initial cases*.

*^b^UP transection was done in nine cases that maintained VC after other maneuvers*.

**Figure 1:**
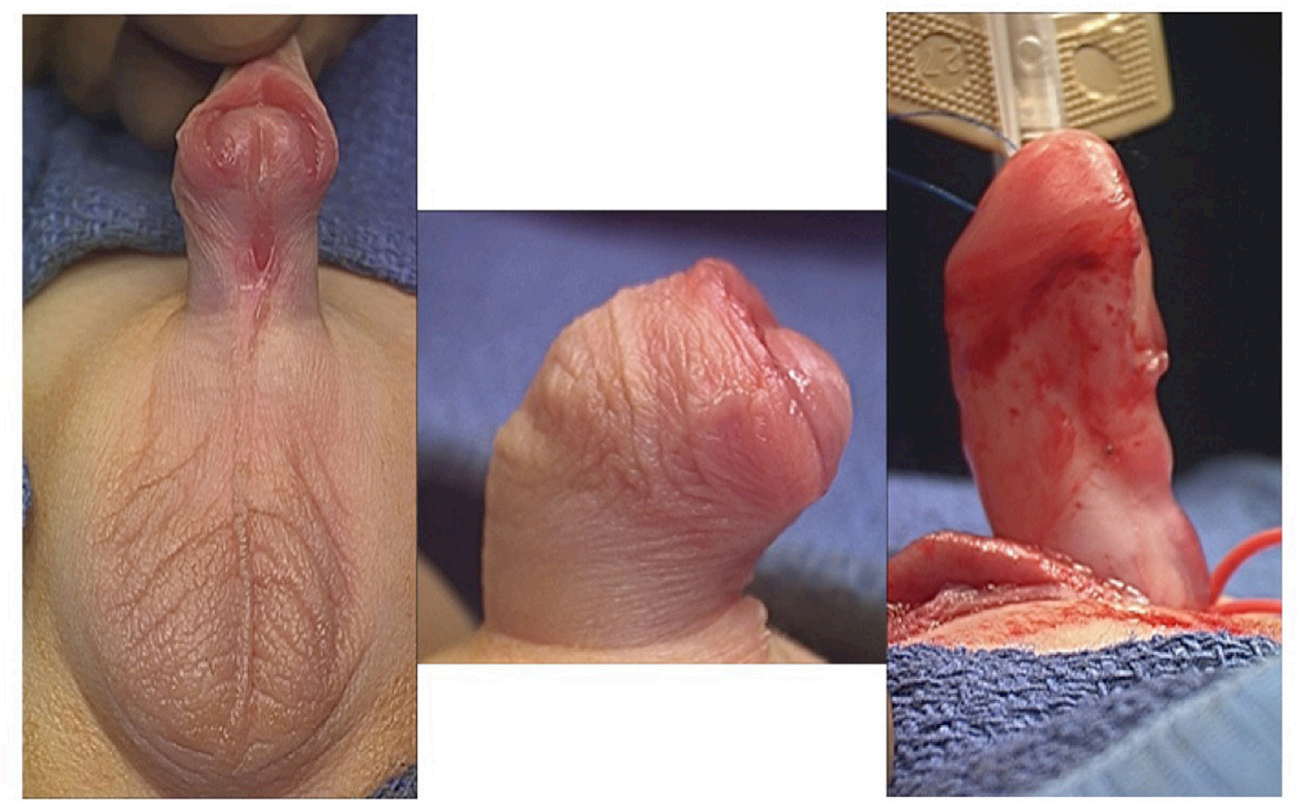
Correction of the curvature after dissection of the penile skin and dartos fascia.

The initial step of the VC repair in patients with hypospadias consists of releasing the skin and fibrotic tissue from the penile dartos. The dissection of these tissues with scissors should be performed down to the base of the penis. This dissection along with the transfer of dorsal penile skin to the ventral part of the penis can properly correct the curvature in most of the mild cases. After performing all these initial maneuvers, an erection test can be performed to assess proper correction of the curvature (Figure 1). Even for proximal hypospadias, degloving the penis alone can correct the VC, and transection of the UP should be avoided before repeating an artificial erection. Weber et al. studied 137 patients with proximal hypospadias and reported that degloving the penis alone was sufficient for VC correction in 7 of 9 (77%) mild cases, 14 of 44 (30%) moderate, and only 2 of 84 (2%) severe cases (25).

## Dorsal Plication Technique

**Figure 2:**
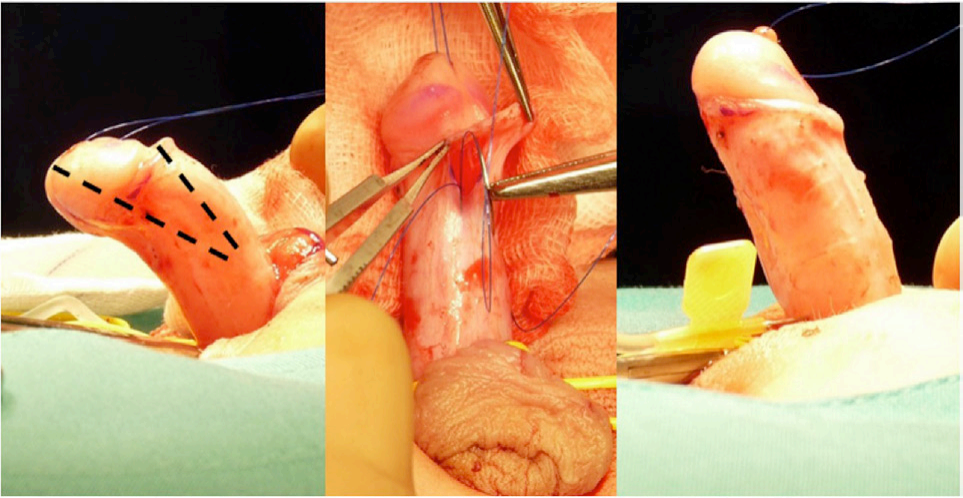
12 O’clock dorsal plication (Baskin) to a low-grade curvature.

The plication principle was first published by Nesbit in 1965, based on the pyloroplasty technique published by Heineke and Mikulicz (27–29) (Figure 2). The procedure included resection of a diamond-shape wedge of dorsal lateral albuginea at the point of maximum curvature and suture of the defect afterward. Several modifications to this technique have been reported, including dorsal plication without resection of the penile fascia (30).

Anatomical studies published by Baskin et al. reported that the best area for plication is at the 12 O’clock position in the dorsal penile aspect (31). This location provides a thicker area of the tunica albuginea and nerves fibers are not found (5). According to his anatomical studies, dissection in the dorsal aspect beyond 1–11 O’clock has a higher risk of injuring the nerve pedicle. The dorsal plication is performed with one or two parallel stitches, and, good results have also been reported by other surgeons (32, 33).

The plication is usually made with two parallel non-absorbable prolene sutures at the point of maximum penile curvature on the dorsal surface of the penis. Some potential disadvantages with the Baskin technique include the presence of dorsal suture (which may be bothersome for some patients) and limited effectiveness when used in older patients as these sutures may not support the pressure exerted by erections in these patients during intercourse (34). In patients with low-grade curvature, recurrence of penile curvature after plication on the dorsal surface was reported in 7% of patients (32).

However, for high-grade curvatures encountered after degloving (>30–40°), the best surgical alternative is not yet defined. There is a concern that the surgical correction using multiple dorsal plications could lead to a high risk of recurrence and reduction of the penile length (35). Chertin et al. reported that 6 of 28 patients (21.4%) who initially underwent dorsal plication surgery had recurrence of VC (36). Consequently, other techniques must be considered if a curvature >30–40° persists after degloving and dissection down to the base of the penis.

A stepwise approach has been proposed by some authors to correct penile curvature (12, 16, 26) with an artificial erection performed next each maneuver (Flowchart). After degloving the penis, an extensive mobilization of the spongiosum/UP followed by a proximal dissection to the bulbar region should be performed in those patients (12, 37–39). Additional steps like simple plication or multiple transverse corporotomies can be added if the curvature still is >30°. Snodgrass and Prieto reported that straightening of the penis and UP preservation could be reached in 85% of cases when all these maneuvers are performed (12). Similarly, Bhat et al. reported that penile curvature could be straightened in 88% of cases after mobilization of the corpus spongiosum/UP and after dissection of the proximal urethra up to the bulbar region. Conversely, Pippi Sale et al. have shown that a VC recurrence rate was significantly higher after mobilization of the UP for correction of VC >30° (20). Preservation of the UP can be performed in selected cases, and in these cases, it is possible to repair the hypospadias with TIP urethroplasty or onlay preputial flaps (when the UP is poor) (26). Moreover, elevation of the UP may be associated with other maneuvers like corporeal rotation (40), multiple transverse corporotomies (“fairy cuts”) (12, 35), or corporotomy and grafting (41).

The division of the UP at the maximum point of curvature is sometimes necessary since the UP can maintain tension, contribute to certain degree of curvature, and shorten the phallus. After this step, a repeat artificial erection should be obtained to ensure degree of curvature. If curvature persists but it is <30°, a plication technique should be considered. If it is still of high grade (>30°), a penile elongation technique is the preferred option for many authors (12, 42, 43). Different types of repairs are included under penile elongation techniques. The idea is to lengthen the ventral aspect of the penis and avoid the use of dorsal plication techniques that risk shortening the phallus. Dorsal plication techniques may lead to length loss with severe VC correction, which is especially concerning in patients with proximal hypospadias, who already have a smaller penis. Braga et al. reported on patients with proximal hypospadias and severe curvature (greater than 45°) that were repaired using dorsal plication versus ventral lengthening techniques (16). Patients were followed up for a mean of approximately 5 years in each group. The UP was transected in 94 and 23.5% of patients with ventral lengthening and with dorsal plication, respectively. Recurrent curvature was seen in 9.4% of the ventral lengthening group compared to 28% of the dorsal plication group.

Some authors advocate the use of multiple transverse incisions on the ventral face of the tunica albuginea (fairy cuts). This technique avoids the use of a graft and it is technically easier to do it. This procedure is performed by making multiples transverse corporotomies at the point of maximum curvature from the 4–8 O’clock positions. The incision is through tunica albuginea until erectile tissues are visible (18). Some recent publications reported the use of grafts for a subsequent urethral tubularization after grafting onto multiple transverse incisions surfaces. Snodgrass and Bush have advocate the use of a 2-stage graft reconstruction after UP transection and multiple transverse corporotomies with an overall graft take of 95% with no difference in those placed onto smooth surfaces (44). In a retrospective review of patients undergoing proximal hypospadias repair, Pippi Salle et al. have demonstrated good outcomes with a staged procedure after multiple deep incisions followed by grafting with a recurrence rate of VC of 4.1% with this technique (20).

**Figure 3:**
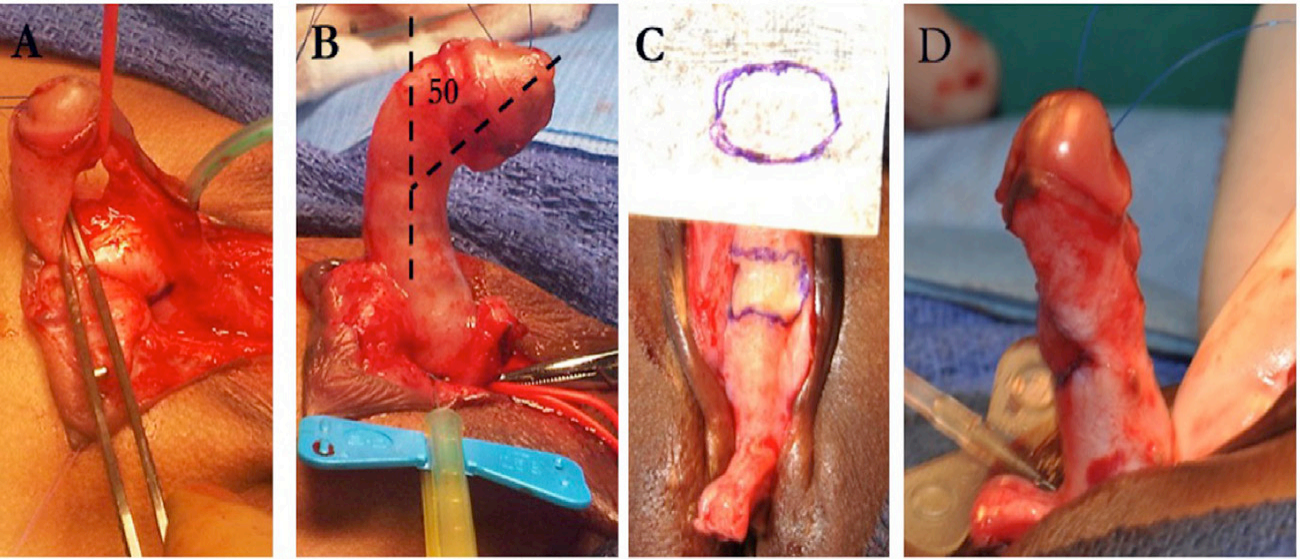
High-grade penile curvature—**(A)** urethral plate elevation; **(B)** measurement of curvature after urethral plate division; **(C)** corporoplasty with small intestine submucosa (SIS) graft; and **(D)** artificial erection after corporoplasty.

Other surgeons prefer to perform a single ventral transverse incision in the tunica albuginea and apply a graft such as tunica vaginalis, intestinal submucosa, small intestine submucosa (SIS) (Figure 3) graft (SURGISIS, Cook Urological, Inc., Indianapolis, IN, USA), or dermis (12, 42). Caution must be taken when making this transverse incision to not injury the nerve supply along the lateral aspect of penis. Usually, this technique is done in the first stage of a 2-stage hypospadias followed by flap techniques (i.e., Byars-flap). Devine and Horton described in 1975 their experience using dermal tissue to the tunica albuginea to correct penile chordee (45). Several other authors have reported similar success results using this technique (46–48).

The SIS graft is an acellular porcine matrix tissue composed mostly of type-1 collagen glycoproteins, proteoglycans, and abundant growth factors. Initial studies in animals followed by humans showed safe and good results with the use of this tissue (49, 50). After applied, the SIS graft produces an initial acute inflammation, followed by a neovascularization process and a cellular growth of native tissue between 3 and 4 weeks. Kropp et al. in an experimental study with rabbits showed that the SIS graft (1-ply) applied in the urethra was completely replaced by well-collagenized tissue, similar to that of normal tunica albuginea after 8–12 weeks (50). El-Assmy et al. compared the use of SIS graft and tunica vaginalis for correction of penile curvature in an animal model. They concluded that although the two tissues are viable options, the SIS graft represented a better option for correction since it was easy to use, with shorter operative time and without morbidity related to donor tissue (49). Our experience in Miami correcting severe penile curvatures in patients with hypospadias was reported in 2011 (42). Between 2001 and 2007, 58 boys underwent ventral corporoplasty using SIS graft (one ply) for severe curvature associated with hypospadias. A two-step correction approach was chosen for 43 patients, while a single step with preputial flap urethroplasty was done in 15 patients. With an average follow-up of 4.8 years, the penile curvature was resolved in 57/58 of the patients. One patient had an additional procedure since an “overcorrection” (dorsal curvature) was noticed postoperatively. A plication on the ventral penile aspect corrected this problem.

Although complications reported with ventral corporoplasty are low, there are still some concerns such as aneurysmal dilatation, penile instability, predisposition to penile fracture, and erectile dysfunction secondary to venous leakage (30). Few publications have reported the postpubertal outcome of patients treated during childhood with any of the VC repair techniques. Badawy and Morsi reported in 2008 a series of 16 patients submitted to corporoplasty with dermal graft (51). In the postpubertal period, 15 of 16 reported good erections with 3 sexually active. One patient presented with erectile dysfunction was treated with intracavernous injections.

Long-term follow-up is required especially for patients with severe proximal hypospadias. Frauman et al. in a study on an online survey with patients submitted to severe hypospadias repair during childhood have found persistence of curvature in 38% of the respondents. These outcomes intuitively can affect the sexual function of these patients. In a long-term study, Chertin et al. evaluated sexual function based on previously validated questionnaires. Of 18 patients who had undergone proximal hypospadias repair, 16 (89%) had erectile dysfunction (13 mild and 3 with moderate erectile dysfunction) and 16 (89%) had premature ejaculation (52). These reports reinforce the necessity of long-term follow-up especially in the postpubertal period to certify correct penile curvature and good sexual function.

## Key Points

**Figure 4:**
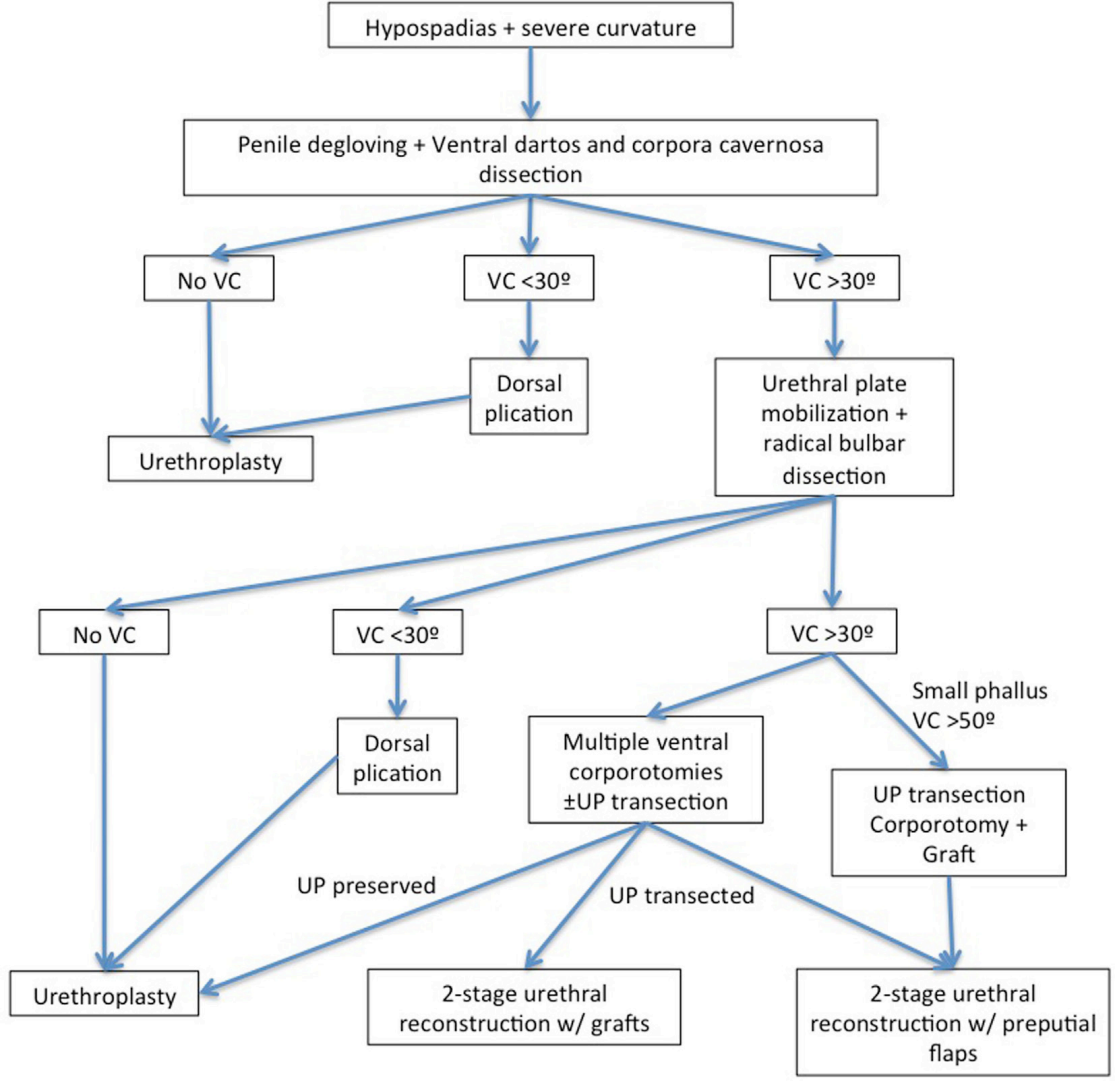
Stepwise approach for the hypospadias associated with high-grade curvature (12, 16, 26).

▪ Penile curvature may represent a complex problem for the pediatric urologist. A better understanding of the anatomy of the penile neurovasculature has led to an improvement in surgical techniques and their results.
▪ Many surgical techniques have been developed (Figure 4); however, none of them are free of complications and studies with longer follow-up, especially in the postpubertal period, are necessary.
▪ Penile curvature can be resolved only by dissecting and degloving the penis. The release of fibrotic tissue and the dartos fascia with scissors should be performed down to the base of the penis. The correction of the curvature is possible with this maneuver and the transfer of dorsal skin to the ventral aspect of the penis (25).
▪ For penile curvatures <30°, the most used technique to correct it is based on the plication of the dorsal albuginea at the point of maximum curvature. Baskin et al. popularized this technique placing stitches at the point of maximum curvature, at hour 12 O’clock of the penile dorsal aspect (31). In this site, the nerve supply is not encountered and the tunica albuginea is thicker. Recurrence rates using this technique in patients with low-grade curvature was reported as 7% (32).
▪ Proximal hypospadias with curvature >30° occur in approximately only 5–10% of cases. The management of patients with high-grade curvature tends to be more complex (Flowchart). Normally, there is a disproportion between the length of the dorsal surface and the ventral aspect of the corpora cavernosa (1). The first surgical step in these patients is the mobilization of the urethra, spongiosum, and all fibrous tissue.
▪ It is sometimes necessary to section the UP at the point of maximum curvature and dissect the proximal, distal urethra and spongiosum to improve penile length and avoid retraction by the short and fibrotic UP.
▪ Penile elongation techniques include execution of multiple transverse incisions on the ventral aspect of the tunica albuginea (fairy cuts) or, performing a single transverse incision and applying a graft of tunica vaginalis, SIS graft, or dermis (12, 42).
▪ A long follow-up after puberty is mandatory in patients submitted to these surgical corrections during all childhood and puberty to secure good and sustained outcomes.

## Author Contributions

PM, manuscript writing, editing, and revision. MC, manuscript writing, editing, and revision. RG, editing and revision.

## Conflict of Interest Statement

The authors declare that the research was conducted in the absence of any commercial or financial relationships that could be construed as a potential conflict of interest.

## References

[1] Baskin LS Duckett JW Lue TF Penile curvature. Urology (1996) 48:347–56. 10.1016/S0090-4295(96)00213-0 8804484

[2] Kramer SA Aydin G Kelalis PP. Chordee without hypospadias in children. J Urol (1982) 128:559–61. 10.1016/S0022-5347(17)53045-1 7120563

[3] Donnahoo KK Cain MP Pope JC Casale AJ Keating MA Adams MC Etiology, management and surgical complications of congenital chordee without hypospadias. J Urol (1998) 160:1120–2. 10.1097/00005392-199809020-00041 9719289

[4] Kaplan GW Lamm DL. Embryogenesis of chordee. J Urol (1975) 114:769–72. 10.1016/S0022-5347(17)67140-4 1185878

[5] Baskin LS Erol A Li YW Cunha GR. Anatomical studies of hypospadias. J Urol (1998) 160:1108–15. 10.1016/S0022-5347(01)62711-3 9719287

[6] Braga LH Pippi Salle JL Dave S Bagli DJ Lorenzo AJ Khoury AE. Outcome analysis of severe chordee correction using tunica vaginalis as a flap in boys with proximal hypospadias. J Urol (2007) 178:1693–7. 10.1016/j.juro.2007.03.166 17707021

[7] Gittes RF McLaughlin AP III Injection technique to induce penile erection. Urology (1974) 4:473–4. 10.1016/0090-4295(74)90025-9 4418594

[8] Bologna RA Noah TA Nasrallah PF McMahon DR Chordee: varied opinions and treatments as documented in a survey of the American Academy of Pediatrics, Section of Urology. Urology (1999) 53:608–12. 10.1016/S0090-4295(98)00656-6 10096392

[9] Springer A Krois W Horcher E. Trends in hypospadias surgery: results of a worldwide survey. Eur Urol (2011) 60:1184–9. 10.1016/j.eururo.2011.08.031 21871708

[10] Fraumann SA Stephany HA Clayton DB Thomas JC Pope JCT Adams MC Long-term follow-up of children who underwent severe hypospadias repair using an online survey with validated questionnaires. J Pediatr Urol (2014) 10:446–50. 10.1016/j.jpurol.2014.01.015 24582083

[11] Tugtepe H Thomas DT Kandirici A Yener S Dagli T. Should we routinely test for chordee in patients with distal hypospadias? Eur J Pediatr Surg (2015) 25:195–8. 10.1055/s-0034-1368797 24683107

[12] Snodgrass W Prieto J. Straightening ventral curvature while preserving the urethral plate in proximal hypospadias repair. J Urol (2009) 182:1720–5. 10.1016/j.juro.2009.02.084 19692004

[13] Ghali AM el-Malik EM al-Malki T Ibrahim AH. One-stage hypospadias repair. experience with 544 cases. Eur Urol (1999) 36:436–42. 10.1159/000020027 10516456

[14] Ferro F Zaccara A Spagnoli A Lucchetti MC Capitanucci ML Villa M. Skin graft for 2-stage treatment of severe hypospadias: back to the future? J Urol (2002) 168:1730–3. 10.1097/00005392-200210020-00018 12352346

[15] Johal NS Nitkunan T O’Malley K Cuckow PM. The two-stage repair for severe primary hypospadias. Eur Urol (2006) 50:366–71. 10.1016/j.eururo.2006.01.002 16464530

[16] Braga LH Lorenzo AJ Bagli DJ Dave S Eeg K Farhat WA Ventral penile lengthening versus dorsal plication for severe ventral curvature in children with proximal hypospadias. J Urol (2008) 180:1743–7. 10.1016/j.juro.2008.03.087 18721961

[17] Ghanem MA Nijman RJ. Outcome analysis of tubularized incised urethral plate using dorsal dartos flap for proximal penile hypospadias repair. J Pediatr Urol (2010) 6:477–80. 10.1016/j.jpurol.2009.11.008 20110195

[18] Snodgrass W Bush N Tubularized incised plate proximal hypospadias repair: continued evolution and extended applications. J Pediatr Urol (2011) 7:2–9. 10.1016/j.jpurol.2010.05.011 20598641

[19] McNamara ER Schaeffer AJ Logvinenko T Seager C Rosoklija I Nelson CP Management of proximal hypospadias with 2-stage repair: 20-year experience. J Urol (2015) 194:1080–5. 10.1016/j.juro.2015.04.105 25963188 PMC4575613

[20] Pippi Salle JL Sayed S Salle A Bagli D Farhat W Koyle M Proximal hypospadias: a persistent challenge. Single institution outcome analysis of three surgical techniques over a 10-year period. J Pediatr Urol (2016) 28:e1–7. 10.1016/j.jpurol.2015.06.011 26279102

[21] Chen C Yang TQ Chen JB Sun N Zhang WP. The effect of staged transverse preputial island flap urethroplasty for proximal hypospadias with severe chordee. J Urol (2016) 196:1536–40. 10.1016/j.juro.2016.05.098 27259652

[22] Long CJ Chu DI Tenney RW Morris AR Weiss DA Shukla AR Intermediate-term followup of proximal hypospadias repair reveals high complication rate. J Urol (2017) 197:852–8. 10.1016/j.juro.2016.11.054 27840122 PMC5462455

[23] Snodgrass W Bush N Staged tubularized autograft repair for primary proximal hypospadias with 30-degree or greater ventral curvature. J Urol (2017) 198(3):680–6. 10.1016/j.juro.2017.04.019 28400187

[24] Lanciotti M Betti M Elia A Landi L Taverna M Cini C Proximal hypospadias repair with bladder mucosal graft: our 10 years experience. J Pediatr Urol (2017) 13:294.e1–294.e6. 10.1016/j.jpurol.2017.01.011 28341425

[25] Weber BA Braga LH Patel P Pippi Salle JL Bagli DJ Khoury AE Impact of penile degloving and proximal ventral dissection on curvature correction in children with proximal hypospadias. Can Urol Assoc J (2014) 8:424–7. 10.5489/cuaj.2337 25553156 PMC4277522

[26] Castagnetti M El-Ghoneimi A. Surgical management of primary severe hypospadias in children: systematic 20-year review. J Urol (2010) 184:1469–74. 10.1016/j.juro.2010.06.044 20727541

[27] Finney JM A new method of pyloroplasty. Bull Johns Hopkins Hosp (1902) 13:155.

[28] Mikulicz J Zur operativen behandlung des stenosirenden magenschwures. Arch Klin Chir (1888) 37:79.

[29] Nesbit RM Operation for correction of distal penile ventral curvature with or without hypospadias. Trans Am Assoc Genitourin Surg (1966) 58:12–4. 5963382

[30] Dason S Wong N Braga LH. The contemporary role of 1 vs. 2-stage repair for proximal hypospadias. Transl Androl Urol (2014) 3:347–58. 10.3978/j.issn.2223-4683.2014.11.04 26813851 PMC4708137

[31] Baskin LS Erol A Li YW Liu WH. Anatomy of the neurovascular bundle: is safe mobilization possible? J Urol (2000) 164:977–80. 10.1097/00005392-200009020-00014 10958721

[32] Bar Yosef Y Binyamini J Matzkin H Ben-Chaim J. Midline dorsal plication technique for penile curvature repair. J Urol (2004) 172:1368–9. 10.1097/01.ju.0000138341.68365.b6 15371846

[33] Yucel S Sanli A Kukul E Karaguzel G Melikoglu M Guntekin E. Midline dorsal plication to repair recurrent chordee at reoperation for hypospadias surgery complication. J Urol (2006) 175:699–702. 10.1016/S0022-5347(05)00186-2 16407031

[34] Shenfeld OZ Re: midline dorsal plication technique for penile curvature repair. J Urol (2005) 173:1830–1. 10.1097/00005392-200505000-00156 15821606

[35] Devine CJ Jr Chordee in hypospadias. In: Glenn J, editor. Urologic Surgery. Philadelphia: JB Lippincott (1983). p. 775.

[36] Chertin B Koulikov D Fridmans A Farkas A. Dorsal tunica albuginea plication to correct congenital and acquired penile curvature: a long-term follow-up. BJU Int (2004) 93:379–81. 10.1111/j.1464-410X.2003.04621.x 14764142

[37] Baker LA Mathews RI Docimo SG. Radical bulbar dissection to correct severe chordee and proximal hypospadias. J Urol (2000) 164:1347–9. 10.1016/S0022-5347(05)67194-7 10992412

[38] Bhat A. Extended urethral mobilization in incised plate urethroplasty for severe hypospadias: a variation in technique to improve chordee correction. J Urol (2007) 178:1031–5. 10.1016/j.juro.2007.05.074 17632146

[39] Mollard P Castagnola C. Hypospadias: the release of chordee without dividing the urethral plate and onlay island flap (92 cases). J Urol (1994) 152:1238–40. 10.1016/S0022-5347(17)32557-0 8072112

[40] Decter RM. Chordee correction by corporal rotation: the split and roll technique. J Urol (1999) 162:1152–4. 10.1097/00005392-199909000-00069 10458453

[41] Kajbafzadeh AM Arshadi H Payabvash S Salmasi AH Najjaran-Tousi V Sahebpor AR. Proximal hypospadias with severe chordee: single stage repair using corporeal tunica vaginalis free graft. J Urol (2007) 178:1036–42. 10.1016/j.juro.2007.05.062 17632178

[42] Castellan M Gosalbez R Devendra J Bar-Yosef Y Labbie A. Ventral corporal body grafting for correcting severe penile curvature associated with single or two-stage hypospadias repair. J Pediatr Urol (2011) 7:289–93. 10.1016/j.jpurol.2011.03.008 21527210

[43] Schlomer BJ. Correction of residual ventral penile curvature after division of the urethral plate in the first stage of a 2-stage proximal hypospadias repair. Curr Urol Rep (2017) 18:13. 10.1007/s11934-017-0659-x 28213855

[44] Snodgrass W Bush N Outcomes of 2-stage graft repair for proximal hypospadias with >30° ventral curvature: modified glansplasty reduces glans dehiscence. ESPU. Prague, Czech Republic (2015).

[45] Devine CJ Jr Horton CE Use of dermal graft to correct chordee. J Urol (1975) 113:56–8. 10.1016/S0022-5347(17)59406-9 1089811

[46] Hendren WH Keating MA. Use of dermal graft and free urethral graft in penile reconstruction. J Urol (1988) 140:1265–9. 10.1016/S0022-5347(17)42020-9 3054166

[47] Lindgren BW Reda EF Levitt SB Brock WA Franco I. Single and multiple dermal grafts for the management of severe penile curvature. J Urol (1998) 160:1128–30. 10.1097/00005392-199809020-00043 9719291

[48] Caesar RE Caldamone AA. The use of free grafts for correcting penile chordee. J Urol (2000) 164:1691–3. 10.1016/S0022-5347(05)67084-X 11025749

[49] El-Assmy A El-Hamid MA Abo-Elghar ME Hafez AT. Single-layer small intestinal submucosa or tunica vaginalis flap for correcting penile chordee. BJU Int (2004) 94:1097–101. 10.1111/j.1464-410X.2004.05110.x 15541135

[50] Kropp BP Ludlow JK Spicer D Rippy MK Badylak SF Adams MC Rabbit urethral regeneration using small intestinal submucosa onlay grafts. Urology (1998) 52:138–42. 10.1016/S0090-4295(98)00114-9 9671888

[51] Badawy H Morsi H. Long-term followup of dermal grafts for repair of severe penile curvature. J Urol (2008) 180:1842–5. 10.1016/j.juro.2008.04.082 18721971

[52] Chertin B Natsheh A Ben-Zion I Prat D Kocherov S Farkas A Objective and subjective sexual outcomes in adult patients after hypospadias repair performed in childhood. J Urol (2013) 190:1556–60. 10.1016/j.juro.2012.12.104 23306088

